# Correlation Analysis Between Japanese Literature and Psychotherapy Based on Diagnostic Equation Algorithm

**DOI:** 10.3389/fpsyg.2022.906952

**Published:** 2022-05-30

**Authors:** Jun Shen, Leping Jiang

**Affiliations:** ^1^School of Foreign Languages, Changshu Institute of Technology, Suzhou, China; ^2^The Institute for Sustainable Development, Macau University of Science and Technology, Macau, China

**Keywords:** equation diagnostic algorithms, attention mechanism, Japanese literature, psychotherapy, Text-CNN, BiGRU

## Abstract

Literary therapy theory provides a new field for contemporary literature research and is of great significance for maintaining the physical and mental health of modern people. The quantitative evaluation of psychotherapy effects in Japanese healing literature is a hot research topic at present. In this study, a text convolutional neural network (Text-CNN) was selected to extract psychological therapy features with different levels of granularity by using multiple convolutional kernels of different sizes. Bidirectional threshold regression neural network (BiGRU) can characterize the relationship between literature research and the psychotherapy effect. On the basis of the CNN-BilSTM model, a parallel hybrid network integrated with the attention mechanism was constructed to analyze the correlation between literature and psychotherapy. Through experimental verification, the model in this study further improves the accuracy of correlation classification and has strong adaptability.

## Introduction

Ryan Idell, a famous contemporary American literary psychologist and critic, particularly analyzed the development of Western literature and psychology from the late nineteenth century to the early twentieth century. After the First World War, the “inward-turning” movement in western literature gradually erased the frontiers between literature and psychology, and the two tended to combine at the edge of the new century. In view of psychologists and psychotherapists, verbal narration and art related to literary narration can play therapeutic roles. Wang Meng, a writer, said, “The rich spiritual life of man [sic] depends not only on psychology but also on literature.” From the perspective of psychoanalysis and humanistic psychology, the psychological principles of literary therapy can be relied on Goh et al. (2018).

The correlation between literature and psychology can be quantitatively evaluated by the attention mechanism. The attention mechanism originated from the study of human vision, and its primary function is to pay attention to the information that is more critical to the current task. Dabek and Caban (2015) introduced the attention mechanism based on RNN when conducting image processing psychotherapy (Chorowski et al., 2015). Chorowski et al. (2015) used the attention-based neuromachine method for the first time to establish a mental state prediction model (Dabek and Caban, 2015). Huang and Sun (2017) used the attention mechanism to dynamically calculate the source language context solved the problem of capturing long-distance relevance in the source language sentences. Bai et al. (2018) proposed a model integrating double-level attention to improve the performance of emotion analysis. Ravi et al. (2020) put forward an interactive attention mechanism for capturing network faults in text classification key characteristics, focusing on the extraction and classification of key characteristics using the BiLSTM and the CNN, as well as the formation of global and local features: the use of attention mechanism failure and failure mode of the key information that is used to fuse the two form classification features. While literature shows that the study of attention mechanisms is mature in long-distance property correlation, there remains a gap in the correlation analysis between literature and psychology.

This study attempted to analyze the correlation between Japanese literature and psychotherapy using the diagnostic equation algorithm model. In the second part of this study, aiming at the CNN's inability to fully and comprehensively extract psychological therapy feature information, the Text-CNN was used to extract psychological therapy features with different levels of granularity by using multiple convolutional kernels of different sizes and to capture the relationship between sentences in Japanese literary works and readers' psychological emotions. BiGRU was used instead of the BiLSTM to capture the emotionally dependent relationship with a large time step distance more accurately and completely. This principle can explore deep semantic information in Japanese literature: simplify model structure and reduce training time. On the basis of the CNN-BilSTM model, we further optimized the attention mechanism by combining the Text-CNN and the BiGRU in parallel so that the model pays close attention to the features of important words, which cause great psychological fluctuations. Finally, a method of correlation analysis between literature and psychology based on the parallel hybrid network and attention mechanism is proposed. In the third part, this study has a detailed introduction of the application of a parallel hybrid network into the attention mechanism model of the whole process, and it completes the relevant parameter selection and comparative experiments. The experimental results verify the effectiveness of the improvement after the classification model of the parallel hybrid network into the attention mechanism, can effectively improve the precision of correlation analysis, and has a strong ability to adapt.

## Research Status of Literary Therapy

The study of literary therapy has been discussed and applied in many fields and more dimensions.

Research at home and abroad is mainly carried out from three aspects: theoretical discussion, literary therapeutic criticism, and social practice.

### Theoretical Discussion Level

Tang studied the therapeutic relationship between witchcraft and myth and witchcraft and literature, respectively (Shao and Cai, 2018). More attention is paid to the exploration of the narrative therapy principle. According to Tang W, narrative therapy externalizes problems and becomes a means of self-recognition to be noticed. Self-healing of creators also provides therapeutic possibilities for recipients, who can get cured by looking at themselves through the eyes of others in literary studies (Ghamisi et al., 2018). Gu added the isomorphism of the literary narrative structure and the “prior structure” of readers' minds (Chen et al., 2020). The dialogue and blending of individual and narrative discourses can help the individual mind achieve balance and surpass the power of generating and solving problems so as to realize the role of literary narrative in constructing the mind. Dai Y. H. also expanded the narrative theory of literary therapy ritual in his doctoral thesis. Fry, Puop, and Ye Shuxian have a common understanding of the common language of narrative and can use plain words to express surging psychological activities (Zhang et al., 2018). In the discussion of literature and literary therapy for the disease, Zhang M. L. thinks that literary therapy is a kind of medical means and original function (Wang et al., 2020). He talks not just about physical illness but also about the whole, holistic experience of illness, subdivided into physical symptoms, metaphorical illness, psychogenic illness, and doctor–patient interpretations of illness. Literature and therapy are separated from each other in the representation of discipline, but confluence occurs in the actual process of therapy. In daily life, through literature sharing or stimulating instinct, we can get recognition, feel better by venting, get self-affirmation, and achieve a balance between physical, psychological, and social functions. In addition, socio-cultural metaphorical therapy is widely used (Kim, 2017). Literary therapy takes responsibility for the process of correspondence between literature and society and also stimulates the courage and vitality of literature. In addition, Zeng H. W. applied literary therapy to the study of online literature, paying particular attention to the comprehensive effect of all media (Chen et al., 2018).

### The Study of Literary Therapeutic Criticism

The metaphorical sociological function of literature therapy and the medical practice functions of physiology and psychology can be interpreted in the practice of literary study. Literature therapy not only has the largest number of papers applied to the practice of literary criticism, including a new perspective on the study of the works of Haruki Murakami and other relevant writers so that the recipients get a new understanding, but it also provides new ideas for the study of other writers and works (Cheung et al., 2017).

### A Fusion of Psychology and Literature and Its Particular Therapeutic Features

At the level of Japanese literature, the research area has further expanded, involving the sacred ritual of new myth and ancient epic, language magic therapy, psychoanalysis, traumatic memory, ecological therapy, feminist language, literary language therapy, among others. These research results fully demonstrate the objective existence and relatively universal effectiveness of the therapeutic function of literature. It also shows that the literary therapy theory has attracted the attention of scholars in various fields of literature research in China. It is worth studying to distinguish the effect of different literary styles (Chen et al., 2019).

### Application Level of Social Practice

The applied research and exploration of literary therapy have made progress. Studies on the application of literary therapy can be divided into the following categories: The first is the social and cultural construction promoted by the government, and the second is that the education department has a great role to play in the prevention and treatment of psychological diseases among students (Nanjun et al., 2019). Huang Mingfang believes that literary therapy also plays a good role in the prevention of psychological diseases caused by social employment and other pressures on college students. Third, literature therapy can be combined with network media culture (Liu et al., 2020). In particular, the whole media has outstanding leap-forward and integration, and the audience scope and treatment scale of literary therapy have been increased to unprecedented levels. Fourth, literary therapy is an important auxiliary reconstruction measure in various disaster events.

To sum up, all kinds of psychological awareness and ability are relatively weak. Literature therapy in mainland China is relatively lacking in sociological therapy, especially in the research direction of combining with computer deep learning theory, which makes it difficult to directly respond to the current social and cultural development situation.

## Theory of Equation Diagnosis Algorithm

### Attention Mechanism

**Figure 1:**
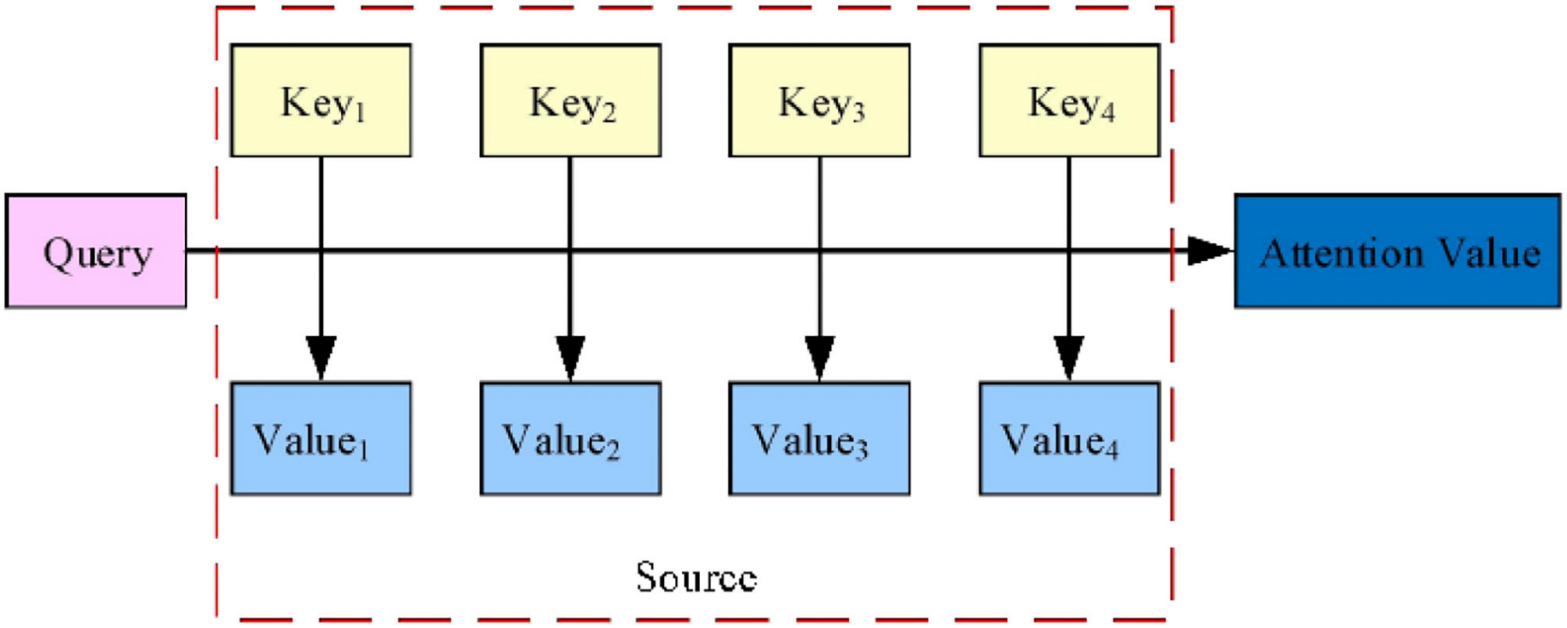
The essence of the attention mechanism.

A traditional machine translation mainly consists of an encoder-decoder architecture, which results in partial information loss and an inability to selectively focus on relevant input sequences as each output information is generated. The encoder-decoder architecture with an attention mechanism can introduce attention weight on the input sequence to give priority to the location set with relevant information to generate the next output information (Xu et al., 2018). By separating the attention mechanism from the encoder-decoder architecture, the essential idea of the attention mechanism can be understood more intuitively, as shown in Figure 1.

As shown in Figure 1, the Source is composed of a series of <*Key, Value*> data pairs. Given a certain element, *Query*, the correlation between *Query* and *Key*_*i*_ is calculated to obtain the corresponding weight coefficient and the weighted sum to obtain the Attention Value. Assuming the length of the Source is *L*, the essential idea of the attention mechanism can be expressed by Formula (1):


(1) $$\begin{array}{l} Attention\left(Query,\;Source\right)=\sum_{i=1}^{L}Similarity\left(Query,\;Key_{i}\right)\;\\ {}*Value_{i} \end{array}$$

**Figure 2:**
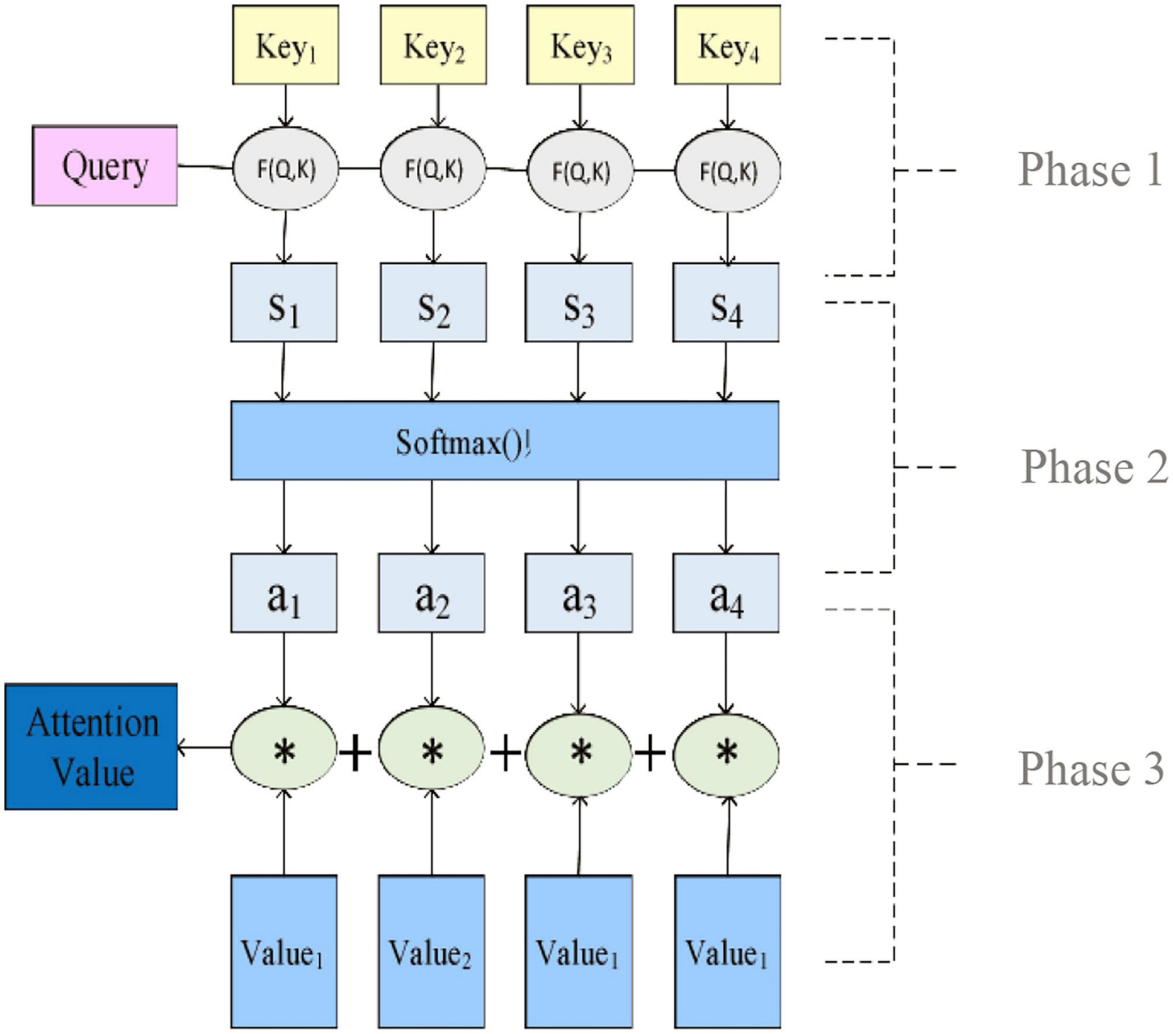
Specific calculation process of attention mechanism.

The specific calculation process of Attention Value can be abstracted into three stages, as shown in Figure 2.

As shown in Figure 2, *Key*_*i*_ is the keyword, *Query* is the sequence *Query, F (Q, K)* is the similarity function, *Value*_*i*_ is the weight, *S*_*i*_ is the correlation calculation *Value* between *Query* and *Key*_*i*_, *a*_*i*_ is the weight coefficient, and Attention Value is the Attention Value finally calculated. In the first stage, different similarity functions and calculation mechanisms are used to calculate the correlation between *Query* and *Key*_*i*_. Commonly used *F (Q, K)* methods include the dot product, matrix multiplication, *cos* similarity, series mode, and multilayer perceptron, as follows:

(1) Dot product

(2) $$\begin{array}{l} S_{i}=F\left(Query,\;Key_{i}\right)=Query^{T}Key_{i} \end{array}$$

(2) Matrix multiplication

(3) $$\begin{array}{l} S_{i}=F\left(Query,\;Key_{i}\right)=Query^{T}W_{a}Key_{i} \end{array}$$

(3) COS similarity

(4) $$\begin{array}{l} \;S_{i}=F\left(Query,\;Key_{i}\right)=\frac{Query^{T}Key_{i}}{\left\|Query\|\cdot \|Key_{i}\right\|} \end{array}$$

(4) Series mode

(5) $$\begin{array}{l} S_{i}=F\left(Query,\;Key_{i}\right)=W_{a}\left[Query;\;Key_{i}\right] \end{array}$$

(5) Multilayer perceptron

(6) $$\begin{array}{l} S_{i}=F\left(Query,\;Key_{i}\right)=v_{a}^{T}\;\tanh\left(W_{a}Query+U_{a}Key_{i}\right) \end{array}$$

The second stage is normalization, highlighting the weight of important elements to obtain the corresponding weight coefficient *a*_*i*_:


(7) $$\begin{array}{l} a_{i}=Soft\text{ max}\left(S_{i}\right)=\frac{e^{S_{i}}}{\sum_{j=1}^{L}e^{S_{j}}} \end{array}$$

In the third stage, the weighted sum yields the Attention Value:


(8) $$\begin{array}{l} Attention\left(Query,\;Source\right)=\sum_{i=1}^{L}a\cdot Value_{i} \end{array}$$

### The Parallel Hybrid Network Into the Attention Mechanism Model Structure

The Text-CNN (Cheng et al., 2019) is an improvement of CNN and a feedforward neural network. Text-CNN extracts emotional features with different levels of granularity and the relationships within and between sentences by using multiple convolution kernels of different sizes. The Bidirectional Gated Recurrent Unit (BiGRU) is mainly composed of GRUs in both positive and negative directions, and the output results are jointly determined by these two GRUs. Not only can BiGRU process the contextual semantic feature information with a large time step distance but the network model structure can also become relatively simple, training parameters are reduced, and the time cost is low. The attention mechanism gives different weights to words with different degrees of importance so that some words get more attention and improve the classification accuracy.

**Figure 3:**
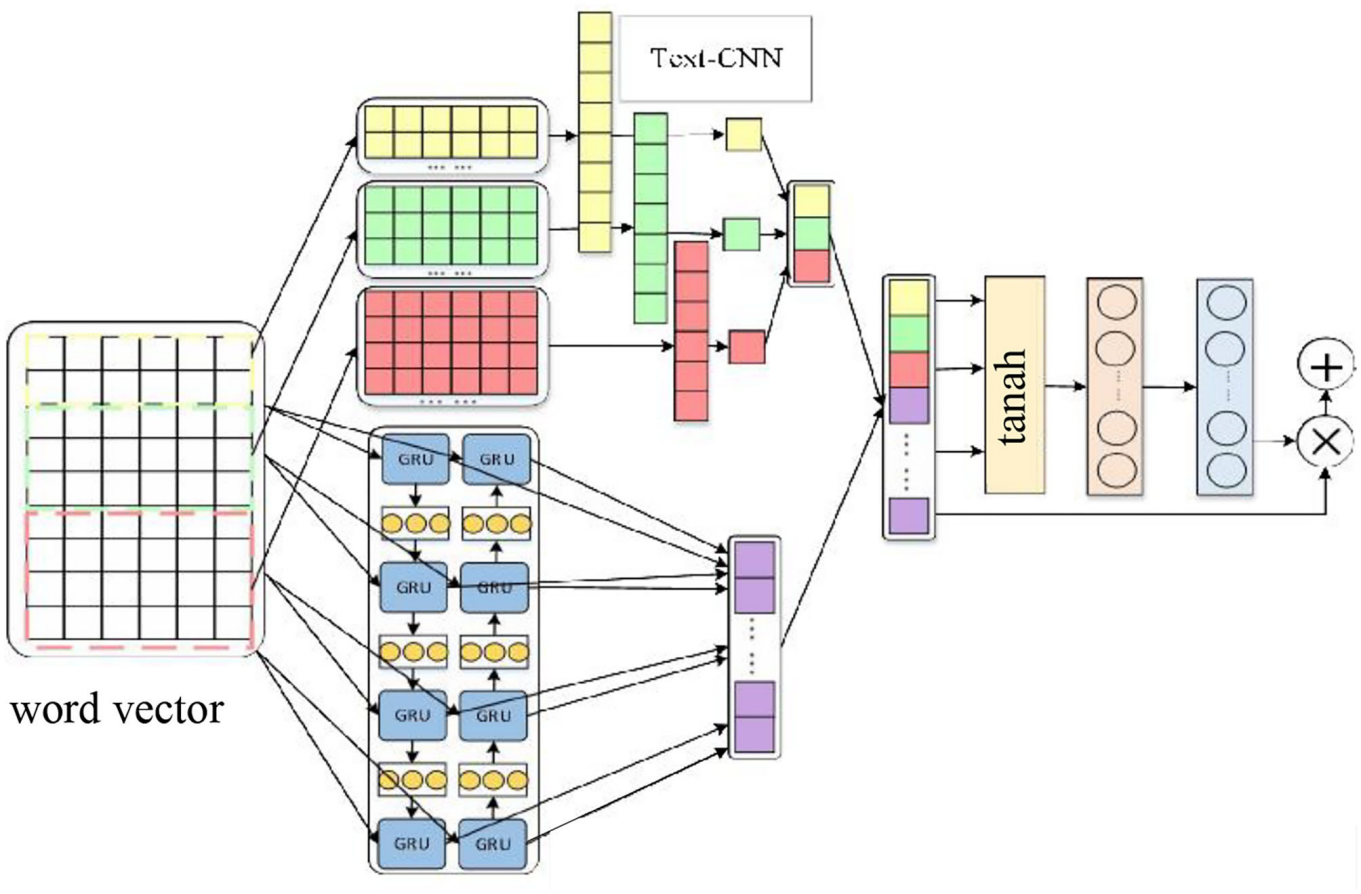
Model based on the parallel hybrid network integrating the attention mechanism.

First, the comment text was vectorized by the GloVe method, and then the Text-CNN and the BiGRU were connected in parallel. Finally, an attention mechanism was introduced to build a new parallel hybrid network and integrate it into the attention mechanism model to solve the related problems of emotion classification, as shown in Figure 3.

#### The Word Vector Represents Text

The word vector text is mainly expressed by mapping the input comment text into the vector form (Dong et al., 2018). The GloVe method is used to map each word in each sentence into the corresponding word vector *x*_*i*_, and then a sentence matrix *S* is formed:


(9) $$\begin{array}{l} S=\left\{x_{1},\;x_{2},\;x_{3},\cdots \cdots \;,x_{n}\right\} \end{array}$$

where $x_{i}\in R^{k}$ and *x*_*i*_are the word vectors of the *i* word, and *k* is the dimension of the word vector. *S* ∈ *R*^*n*×*k*^, and *n* is the number of words. In this study, the GloVe method is used to vectorize the text. Since the GloVe model includes two training methods, namely, global statistical information from matrix decomposition and the local window context of Word2vec. The word vector obtained contains more associated semantic and grammatical information and is superior to the Word2vec word vector method in overall performance.

#### Text—The CNN Layer

The sentence matrix *S* is taken as the input of the Text-CNN layer. By using multiple convolution kernels of different sizes, Text-CNN extracts emotional features with different levels of granularity and the relationship between sentences so as to obtain more comprehensive feature information (Titos et al., 2018).

At the convolution layer, *r* × *k* convolution is used to perform a convolution operation on the sentence matrix S to obtain local semantic information. After passing through the convolution layer, the local feature obtained by the *i* neuron is expressed as *C*_*i*_, *w* is the convolution kernel, *b* is the bias term, *x*_*i*:*i*+*r*−1_ is a total r row vector from *i* to *i* + *r-1* in the sentence matrix, and the calculation of *c*_*i*_ is shown in formula (10):


(10) $$\begin{array}{l} c_{i}=RELU\;\left(w\cdot X_{i:i+r-1}+b\right) \end{array}$$

The pooling layer reduces the dimension of the local semantic features obtained by the convolution operation to capture the optimal solution of the local features. In this chapter, the maximum pooling method is used to extract the maximum feature from the local feature set to replace the whole local feature, as shown in Formula (11):


(11) $$\begin{array}{l} z_{r}=\max\left(c_{1},c_{2},c_{3},\cdots \cdots \;,c_{n-r+1}\right) \end{array}$$

In this chapter, multiple convolution kernels of sizes 3, 4, and 5 are used for the convolution operation, and the obtained characteristic information is input into the pooling layer to obtain three maximum pooling results. The three pooling results are spliced together, and the final output of Text-CNN represents *h*_*T*_ as the input of the attention mechanism layer.


(12) $$\begin{array}{l} h_{T}=z_{3}⊕z_{4}⊕z_{5} \end{array}$$

#### The BiGRU Layer

The Bi-lstm is composed of two lstms with opposite directions. It can not only obtain the above information but also consider the impact of the following information on the current word. If the time step distance is large, the BiLSTM cannot fully obtain the context information of the word. In this paper, the BiGRU is used to solve this problem; it consists of a one-way forward propagation GRU and a one-way backward propagation GRU, and the output results are jointly determined by the GRU in these two directions. The BiGRU can not only fully extract the above information but can also take into account the influence of the following information on the current word, capture the word dependence relationship with a large time step distance, and mine the deep semantic information in the text. Moreover, the network model structure becomes relatively simple, the training parameters are reduced, and the time cost is relatively low. The sentence matrix *S* is used as the input of the BiGRU layer to extract contextual semantic feature information with a large time step distance. The specific processing process of the BiGRU is shown in Formulas (13)–(15):


(13) $$\begin{array}{l} {\vec{h}}_{t}=\sigma \left(W_{x\vec{h}}x_{t}+W_{\vec{h}\vec{h}}{\vec{h}}_{t-1}+b_{\vec{h}}\right) \end{array}$$

(14) $$\begin{array}{l} {\overset{\leftarrow }{h}}_{t}=\sigma \left(W_{x\overset{\leftarrow }{h}}x_{t}+W_{\overset{\leftarrow }{h}\overset{\leftarrow }{h}}{\overset{\leftarrow }{h}}_{t-1}+b_{\overset{\leftarrow }{h}}\right) \end{array}$$

(15) $$\begin{array}{l} y_{B}=W_{hy}\left[{\vec{h}}_{n},{\overset{\leftarrow }{h}}_{i}\right]+b_{y} \end{array}$$

where ${\vec{h}}_{t}$ ${\overset{\leftarrow }{h}}_{t}$is the output of GRU propagating forward and backward, respectively; *y*_*B*_ is the output of BiGRU; *W* is the weight matrix; *b* is the bias vector; and σ is the sigmoid function.

#### Attention Mechanism Layer

**Figure 4:**
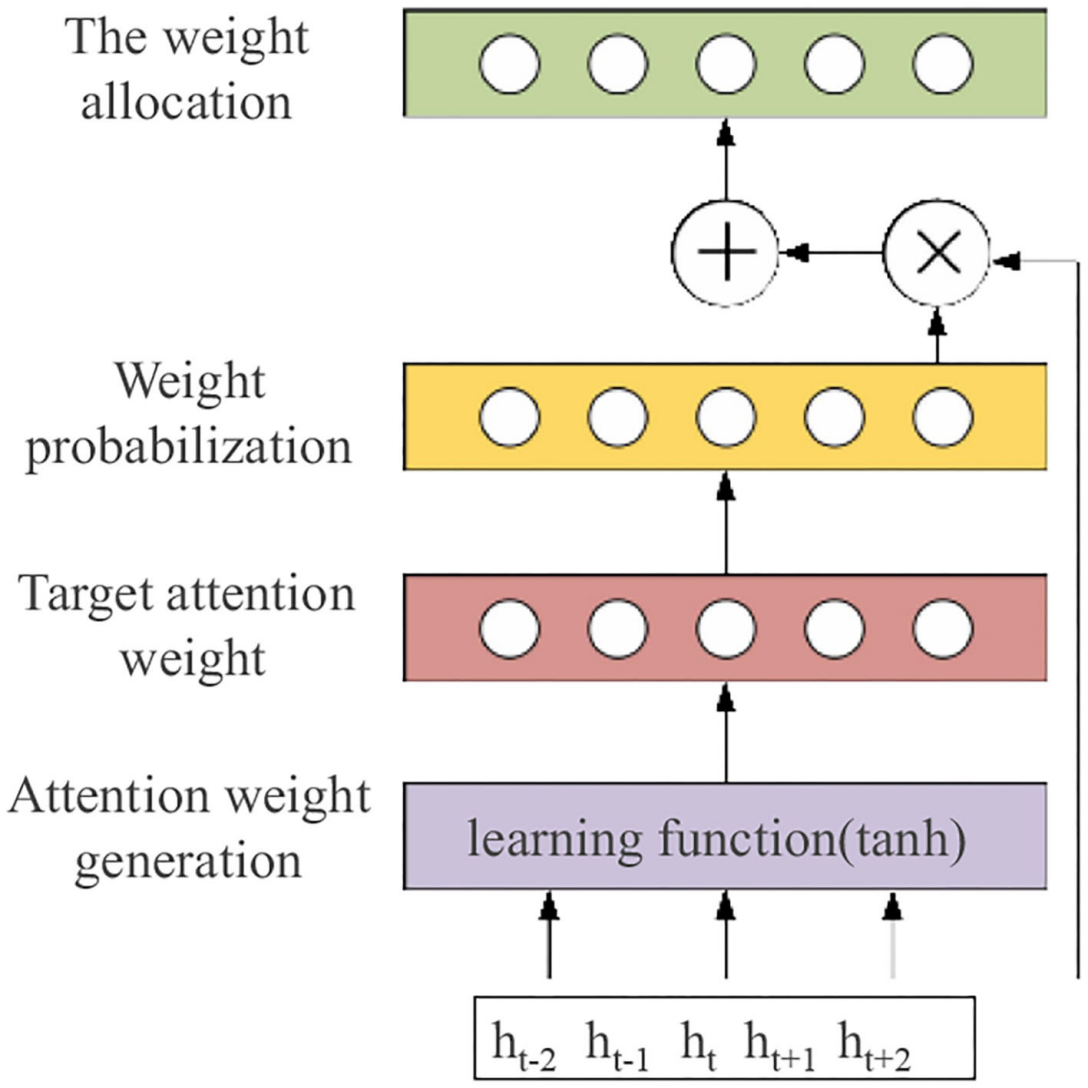
The attention mechanism model structure.

The application of the attention mechanism in text sentiment analysis can calculate the probability weights of different words by their weight distribution so that some words can get more attention, highlight important words, and improve the accuracy of classification. The input of the attention mechanism layer in this chapter is the result of feature fusion extracted by the Text-CNN and the BiGRU, and the weight configuration of the attention distribution of input information to the emotion vector generated by the attention mechanism is calculated. The model structure of the attention mechanism is shown in Figure 4.

(1) The features extracted by the Text-CNN and the BiGRU are fused as the input of the attention mechanism layer, denoted as *h*_*A*_, as shown in Formula (16):

(16) $$\begin{array}{l} h_{A}=h_{T}+y_{B} \end{array}$$

(2) We calculated the target attention weight $u_{t}^{i}$, with *W*_*u*_ as the weight matrix and *b*_*u*_ as the bias vector, as shown in Formula (17):

(17) $$\begin{array}{l} u_{t}^{i}=v_{t}\tanh\left(W_{u}h_{A}+b_{u}\right) \end{array}$$

(3) Normalized by the softmax function, the weight coefficient $a_{t}^{i}$ is obtained by the probability of attention weight so as to highlight the weight of important words, as shown in Formula (18):

(18) $$\begin{array}{l} a_{t}^{i}=soft\text{ max}\left(u_{t}^{i}\right)=\frac{\exp\left(u_{t}^{i}\right)}{\overset{n}{\underset{i=1}{\sum }}\exp\left(u_{t}^{i}\right)} \end{array}$$

(4) Attention weight configuration mainly sum the fused feature *h*_*A*_ and weight coefficient $a_{t}^{i}$ to obtain the text vector *c*^*t*^ of the important information of each word in the text so that the attention weight generated by the model plays an important role, as shown in Formula (19):

(19) $$\begin{array}{l} c^{t}=\overset{n}{\underset{i=1}{\sum }}a_{t}^{i}h_{A} \end{array}$$

#### Emotion Classification Layer

The emotion classification layer takes the text feature of the important information of each word obtained by the attention mechanism layer as input and obtains the final emotion classification result through the sigmoid function.


(20) $$\begin{array}{l} h_{c}=W_{c}c^{t}+b_{c} \end{array}$$

(21) $$\begin{array}{l} P_{c}=\frac{1}{1+\exp\left(-h_{c}\right)} \end{array}$$

where *p*_*c*_ is the probability of a certain class, *W*_*c*_ is the weight matrix, and *b*_*c*_ is the bias vector.

## Experimental Study

### Experimental Data Set and Environment

In order to verify the performance of the parallel hybrid network-integrated attention mechanism model in the correlation analysis of the influence of Japanese literature and psychotherapy, the following two English data sets were mainly selected for relevant experiments, as shown in Table 1.

**Table 1:** Data of the experiment in this chapter.

**Data set name**	**Training set**	**Validation set**	**Test set**	**Total number**
IMDB	32,000	8,000	10,000	50,000
SST-2	8,544	1,101	2,210	11,855

*SST-2: This data set is an extension of Stanford University's MR data set on film reviews. Positive comments are labeled 1, negative comments are labeled 0, and it contains 11,855 texts labeled with emotion classification*.

### Setting and Optimization of Experimental Parameters

Experimental parameter settings directly affect the classification effect of the model. After each iteration, the set hyperparameters are adjusted according to the evaluation index of the experiment. After several iterations, the specific parameter settings are shown in Table 2.

(1) Word vector dimension selection

Select word vectors of 50, 100, 200, and 300 dimensions to explore the influence of word vectors of different dimensions on correlation analysis. Table 3 shows the specific experimental results.

**Table 2:** Parameter setting of the attention mechanism model in the parallel hybrid network.

**The parameter meaning value**	**Name**	**The parameter**
Embedding_dim	Word vector dimension	50, 100, 200, and 300
Filters	Convolution kernel number	32
Kernel size	Convolution kernel size	3,4,5
Padding	Fill the way	Same
Activate on	The activation function	Relu
Pooling size	Pool layer size	2.
BGRU (units)	BGRU Number of hidden layer nodes	32, 64, 96, 128, 160, and 192
Dropout	Random discard probability	0.1, 0.2, 0.3, 0.4, 0.5, 0.6, and 0.7
Dense dim	Number of neural units in full connection layer	100
Batch size	Batch training sample number	16, 32, 64, 128, and 256
Epochs	Practice choosing the number of time steps	10
Optimizer	The optimizer	SGD, Adagrad, Adadelta, RMSProp, and Adam

**Table 3:** Experimental results of the different vector dimensions of words.

**IMDB**					**SST-2**			
**Vector dimension**	**Accuracy**	**Accurate rate**	**Recall rate**	**F1**	**Accuracy**	**Accurate rate**	**Recall rate**	**F1**
50	88.37	88.46	88.27	88.35	88.18	88.43	87.95	88.19
100	89.82	90.67	88.77	89.69	88.61	88.69	88.57	88.63
200.65	91.46	91.36	89.48	88.51	92.32	90.45	90.8	189
300	91.10	93.36	88.42	90.78	88.48	89.80	86.88	88.32

By analyzing Table 3, it can be seen that, when the dimension of the word vector is 50, the accuracy, accuracy rate, recall rate, and F1 of the model on IMDB and SST-2 are the lowest values, indicating that the performance of the model is poor. With the increase of the word vector dimension, the overall performance of the model is improved gradually. When the dimension of the word vector is 200, the accuracy, recall rate, and F1 of the model on IMDB and SST-2 reach their highest values, which are 91.46, 90.45, and 91.36% and 89.48, 90.81, and 89.65%, respectively. When the word vector dimension continues to increase, all the evaluation indexes of the model begin to show a downward trend except for the accuracy. Therefore, in the experiment of this chapter, the dimension of the word vector is set as 200 on IMDB and SST-2.

(2) The selection of the node number of the BiGRU hidden layer

In the experiment of selecting the number of nodes in the BiGRU hidden layer, the number of nodes in the BiGRU hidden layer was set to 32, 64, 96, 128, 160, and 192, respectively, to explore the influence of different numbers of nodes on the results of correlation analysis. Table 4 lists the specific experimental results.

**Table 4:** Experimental results of the different numbers of hidden layer nodes.

**IMDB**					**SST-2**			
**Node number**	**Accuracy**	**Accurate rate**	**Recall rate**	**F1**	**Accuracy**	**Accurate rate**	**Recall rate**	**F1**
32	90.20	92.83	87.09	89.81	88.35	91.83	84.16	87.83
64	90.80	91.68	89.73	90.67	89.10	89.47	88.69	89.08
96	91.46	92.32	90.455	91.36	89.48	88.51	90.81	89.65
128	91.39	90.49	92.57	91.34	89.11	88.71	89.68	89.19
160	91.37	91.81	90.86	91.32	90.82	88.89	86.56	88.64
192	90.95	91.29	90.55	90.90	88.21	87.17	89.73	88.43

Table 4 shows that, when the number of nodes in the BiGRU hidden layer is set to 32, the performance of the model is relatively poor in IMDB and SST-2 because the correlation information cannot be completely extracted as the number of nodes is too small. As the number of nodes continues to increase, the evaluation indexes of the model generally show a trend of first increasing and then decreasing. When the number of nodes in the hidden layer is increased to 96, the accuracy, recall rate, and F1 of the model on the two datasets reach their best values. Therefore, the number of nodes in the BiGRU hidden layer is set to 96 on IMDB and SST-2 in this study.

(3) Selection of the Dropout value

The Dropout values were set to 0.1, 0.2, 0.3, 0.4, 0.5, 0.6, and 0.7 to explore the impact of different Dropout values on correlation analysis results. Table 5 lists the specific experimental results.

**Table 5:** Experimental results of different Dropout values.

**IMDB**					**SST-2**			
**Dropout**	**Accuracy**	**Accurate rate**	**Recall rate**	**F1**	**Accuracy**	**Accurate rate**	**Recall rate**	**F1**
0.1	90.03	90.02	90.04	90.03	87.38	87.64	85.79	86.71
0.2	90.27	90.25	90.31	90.28	87.56	88.65	86.15	87.38
0.3	90.93	90.87	90.97	90.92	88.60	89.42	88.03	88.72
0.4	91.02	90.78	90.06	90.42	89.07	89.36	88.78	89.07
0.5	91.46	92.32	90.45	91.36	89.48	88.51	90.81	89.65
0.6	89.81	89.88	89.71	89.79	88.46	87.87	89.28	88.57
0.7	88.46	88.63	88.48	88.55	88.28	87.16	89.95	88.53

By analyzing Table 5, it can be seen that, when the Dropout value is 0.1, the accuracy, accuracy rate, recall rate, and F1 of the model on IMDB and SST-2 data sets are all the lowest, which may be due to the small number of randomly discarded neurons and too many parameters in the model. The Dropout values continue to increase, and the values of each evaluation index of the model tend to increase. When the Dropout value reaches 0.5, the model's four evaluation indexes on the two datasets reach optimal values. As we continued to increase the Dropout value, the model's learning ability became worse due to a large number of discarded neurons, and the model's performance began to gradually decline, so we set the Dropout value to 0.5 on both datasets.

(4) Batch_size value selection

In the batch sample size selection experiment, this section will set the batch_size values to 16, 32, 64, 128, and 256, respectively, to explore different batches, the influence of size value on the results of correlation analysis. Table 6 lists the specific experimental results.

**Table 6:** Experimental results of different batch_size values.

**IMDB**					**SST-2**			
**Batch size**	**Accuracy**	**Accurate rate**	**Recall rate**	**F1**	**Accuracy**	**Accurate rate**	**Recall rate**	**F1**
16	91.11	90.49	90.12	91.30	88.03	87.31	89.14	88.22
32	91.46	92.32	90.45	91.36	89.48	88.51	90.81	89.65
64	90.61	91.95	88.95	90.43	87.78	89.75	85.34	87.49
128	90.86	92.46	88.97	90.68	86.59	92.15	79.69	85.47
256	88.12	90.10	85.41	87.69	85.55	86.37	84.40	85.37

As can be seen from Table 6, with the continuous increase of batch_size, the accuracy of the model generally increases first and then decreases. When batch_size is 32, the model not only achieves the highest accuracy, recall rate, and F1 value on the IMDB and SST-2 but also achieves a better convergence effect in shorter training time and smaller memory resource occupancy. Therefore, the batch_size value is set to 32 on both IMDB and SST-2.

(5) Selection of the optimizer

For the comparison experiment of optimizer selection, five commonly used optimizers were selected: SGD, Adagrad, Adadelta, RMSProp, and Adam, in order to explore the influence of different optimizers on correlation analysis results. Table 7 lists the specific experimental results.

**Table 7:** Experimental results of different optimizers.

**IMDB**					**SST-2**			
**Optimizer**	**Accuracy**	**Accurate rate**	**Recall rate**	**F1**	**Accuracy**	**Accurate rate**	**Recall rate**	**F1**
SGD	78.83	79.36	77.87	78.61	87.32	87.11	87.77	87.44
A dagrad	87.92	94.84	80.19	86.90	88.25	90.98	84.67	87.71
Adadelta	89.63	89.65	89.61	89.63	88.42	90.42	85.93	88.12
RMSProp	91.22	89.56	90.16	89.86	88.89	87.09	90.65	88.83
Adam	91.46	92.32	90.45	91.36	89.48	88.51	90.81	89.65

As can be seen from the experimental results in Table 7, the Adam optimizer has the best experimental results, and the overall performance of the model is the best, mainly because Adam can make an adaptive adjustment to the learning rate parameters, and the selection of super-parameters is quite robust, so the optimizer selects Adam.

Following the experimental exploration and analysis of the above important parameters, it is concluded that the final hyperparameter settings of the parallel hybrid network integrating attention mechanism model proposed in this study are as follows: the word vector dimension is 200; the node number of the BiGRU hidden layer is 96; the Dropout value is 0.5; batch_size 32; and the optimizer selects Adam. Other parameters are the same as those shown in Table 2.

### Experimental Results and Analysis

#### Contrast Experimental Settings

The correlation analysis method of the influence of Japanese literature and psychotherapy based on a parallel hybrid network and attention mechanism proposed in this chapter will be compared with the following commonly used models to verify the validity of the model presented in this study. In order to ensure the accuracy and comparability of the comparison experiment results, all parameter settings of the comparison experiment are consistent with those of the model experiment proposed in this study.

(1) CNN: A feedforward neural network with a deep structure and convolution computation in the deep learning model is used to extract text phrase features and semantic information.
(2) Text-CNN: An improvement on the CNN, which uses convolution kernels of different sizes to extract emotional features with different levels of granularity and the relationships within and between sentences.
(3) BiLSTM: It is composed of two LSTM in opposite directions, which takes into account the influence of psychological therapy information on current words while obtaining Japanese literature information.
(4) BiGRU: The BiGRU is mainly composed of GRUs in both positive and negative directions. It can not only process literature and psychological-characteristic information with a large time step distance but also has a relatively simple network model structure, reduced training parameters, and a low time cost.
(5) Text-CNN-BiGRU: In critical literature, first, the GloVe method was used to obtain the representation of literary features, and then the Text-CNN network was used to extract the characteristic information of psychotherapy with different levels of granularity, which was used as the input of the BiGRU network model to obtain the information of Rien literature and psychotherapy with a large time step distance through BiGRU. Finally, the sigmoid function is used to obtain the final correlation classification results.
(6) Text-CNN + BiGRU: In critical literature, the GloVe method was first used to obtain the representation of literary features, which were input into Text-CNN and BiGRU networks, respectively, to extract different feature information. Then, the obtained features were segmented and input into the psychotherapy classification layer to obtain the final correlation classification result through the sigmoid function.
(7) Text-CNN + BiGRU + Attention: The correlation analysis method of the parallel hybrid network integrating attention mechanism was proposed in this chapter. First, the GloVe method was used to map critical literature into word vectors and input them into the Text-CNN to extract psychological therapy features with different levels of granularity as well as internal and interrelationships between them, and the feature information of Japanese literature and psychotherapy was extracted from the BiGRU. Then, the features obtained by the Text-CNN and the BiGRU are combined and input into the attention mechanism to assign different weights so that the important words get more attention. Finally, the sigmoid function is used to obtain the final correlation category label.

#### Experimental Results and Analysis

Table 8 shows the experimental results of the model in this chapter and other comparison models on the same data set.

**Table 8:** Experimental results of different models.

**IMDB**					**SST-2**			
**Model**	**Accuracy**	**Accurate rate**	**Recall rate**	**F1**	**Accuracy**	**Accurate rate**	**Recall rate**	**F1**
CNN	86.32	86.33	86.35	86.34	85.49	85.56	85.65	85.60
Text-CNN	87.51	87.41	87.68	87.54	86.22	86.21	87.85	87.02
BiLSTM	90.06	89.99	90.18	90.08	87.94	89.80	85.30	87.49
BiGRU	90.46	89.45	90.86	90.15	88.05	86.68	90.05	88.33
Text-CNN-BiGRU	90.48	90.46	90.50	90.48	88.60	86.94	90.43	88.65
Text-CNN+BiGRU	90.78	90.83	90.67	90.78	88.96	88.99	89.05	89.02
Text-CNN+BiGRU+Attention	91.46	92.32	90.45	91.36	89.48	88.51	90.81	89.65

Table 8 shows seven experimental results. In this chapter, the accuracy, accuracy rate, and F1 values of the Text-CNN + BiGRU + Attention model in the IMDB data set are 91.46, 92.32, and 91.36%, respectively, which are superior to the other six models. The accuracy rate, recall rate, and F1 value of the SST-2 data set reached 89.48, 90.81, and 89.65%, respectively, which were higher than those of the other six models. From groups 1 and 2, it can be seen that the effect of the Text-CNN with multiple convolutional kernels of different sizes is higher than that of single convolutional kernels. From groups 2 and 3, it can be seen that the effect of extracting emotion feature information of different levels of granularity using only the Text-CNN is worse than that of extracting Japanese literature feature information by the BiLSM alone. From groups 3 and 4, it can be seen that the BiGRU with an improved BiLSM structure not only greatly reduces the training time but also improves the model accuracy, recall rate, and F1 to some extent, indicating that BiGRU is more suitable for dealing with correlation tasks than BiLSM. It can be seen from groups 5, 6, 2, 3, and 4 that any combination of the Text-CNN and BiGRU network models is better than a single Text-CNN, BiLSTM, and BiGRU models in the task of correlation analysis. The main reason is that Text-CNN convolution kernels of different sizes can be used to extract text correlation information of different levels of granularity. The BiGRU can extract the learning ability of serialized features, literature, and psychotherapy. Therefore, it has a positive effect on the reprocessing of features extracted by the Text-CNN in a model combination. The chain fusion in group 5 mainly comes from the chain fusion in the Text-CNN and the BiGRU. It can see from Table 5 and Group 6 above that the parallel fusion method proposed in this chapter is better than the chain method in dealing with correlation analysis tasks. The results show that the attentional mechanism can effectively improve the overall performance of the fusion model. The attention mechanism calculates the probability weight of different words through weight distribution, which is conducive to the model grasping essential features and quickly improving classification accuracy.

**Figure 5:**
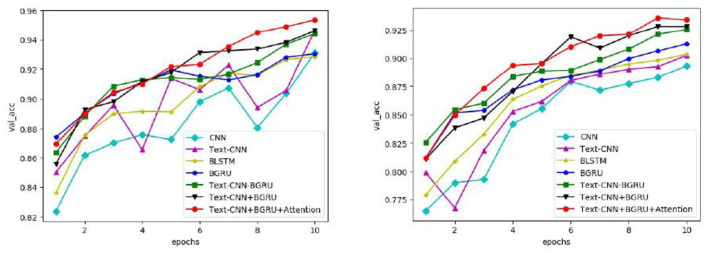
Variation in the accuracy of the verification set for different models.

**Figure 6:**
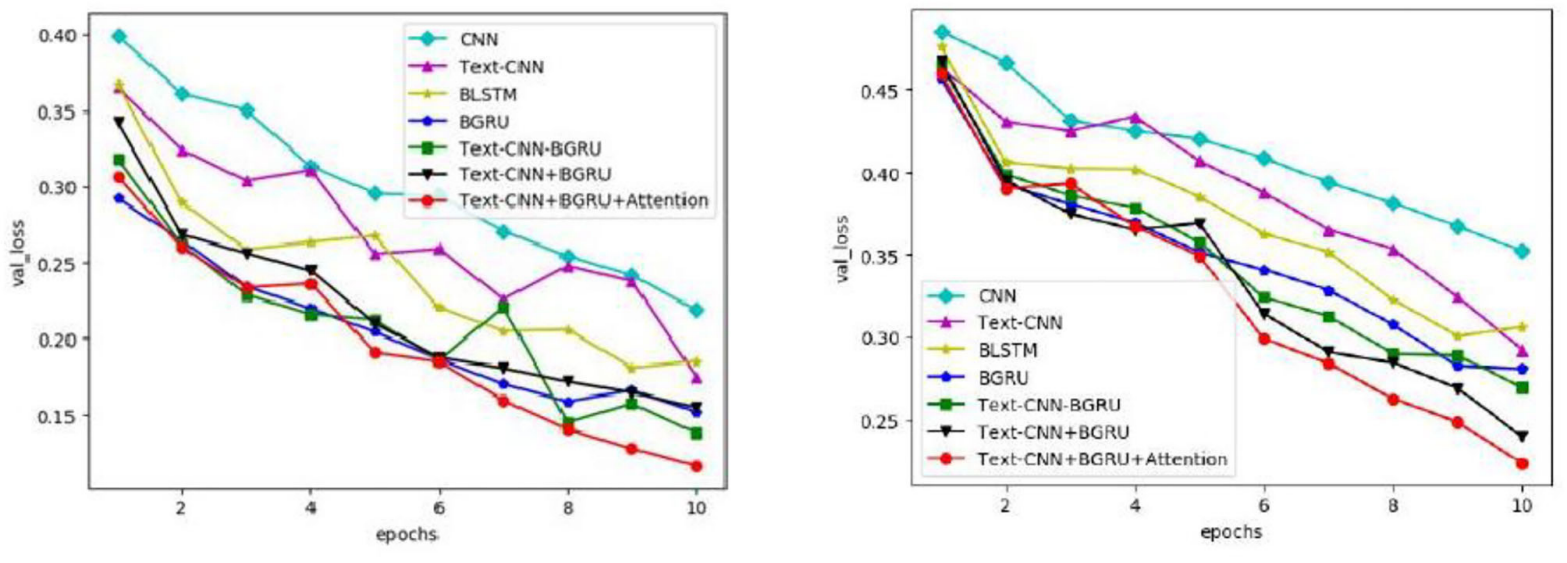
Variation of loss of the verification set for different models.

In this paper, seven commonly used models are used on the IMDB and SST-2 data sets to obtain the changes in accuracy, loss rate, and iteration times of validation sets on different data sets by means of parameter training, as shown in Figures 5, 6, respectively.

As can be seen from Figure 5, with the increase in iteration times, the accuracy of all models gradually increases. In the second iteration, the accuracy of all models on IMDB and SST-2 exceeds 86 and 75%, respectively. Compared with the other six curves, the curve of the model in this study has relatively stable changes and small fluctuations, and the accuracy of the verification set is in a higher position, especially after the seventh iteration, which reaches more than 93% on IMDB and more than 90% on SST-2. It is concluded that the model proposed in this chapter is more stable in the task of extracting the characteristic correlation between literary factors and psychotherapy.

## Conclusion

This chapter proposes a correlation analysis method for Japanese literature and psychotherapy based on the parallel hybrid network integrating the attention mechanism. First, the concrete structure of the parallel hybrid network integrating the attention mechanism model is introduced, which mainly includes the word vector representation literature layer, the Text-CNN layer, the BiGRU layer, the attention mechanism layer, and the correlation classification layer. The network structure and specific operating principles of each layer are further introduced in detail. Then, we selected five groups of important parameters, namely word vector dimension, the node number of the BiGRU hidden layer, the Dropout value, batch_size value, and optimizer, to explore the influence of different parameter values on correlation classification in the IMDB and SST-2 data sets so as to select the experimental parameter values most suitable for the model in this study. Finally, the correlation analysis method between Japanese literature and psychotherapy based on a parallel hybrid network and attention mechanism proposed in this study is compared with several commonly used models on the IMDB and SST-2 data sets. By analyzing the accuracy, accuracy rate, recall rate, and F1 of different models, it is found that the model proposed in this study has the best comprehensive performance in correlation classification and then proves that the parallel hybrid network-integrated attention mechanism model proposed in this study is more suitable for the task of multifactor correlation analysis. The experimental results verifired that the improved parallel hybrid network classification model incorporating an attention mechanism can effectively improve the classification accuracy of the model and has strong application ability.

## Data Availability Statement

Publicly available datasets were analyzed in this study. This data can be found here: https://library.stanford.edu/research-data-services.

## Ethics Statement

The studies involving human participants were reviewed and approved by the Ethics Committee of Changshu Institute of Technology. Written informed consent was obtained from all participants for their participation in this study.

## Author Contributions

JS developed the concept, prepared the figures, and wrote the manuscript. LJ read, revised, and approved the manuscript. Both authors contributed to the article and approved the proof for publication.

## Conflict of Interest

The authors declare that the research was conducted in the absence of any commercial or financial relationships that could be construed as a potential conflict of interest.

## Publisher's Note

All claims expressed in this article are solely those of the authors and do not necessarily represent those of their affiliated organizations, or those of the publisher, the editors and the reviewers. Any product that may be evaluated in this article, or claim that may be made by its manufacturer, is not guaranteed or endorsed by the publisher.
